# Introduction for special issue on pain in developing countries

**DOI:** 10.1097/PR9.0000000000000800

**Published:** 2019-12-06

**Authors:** Daniel Ciampi de Andrade

**Affiliations:** a Department of Neurology, Pain Center, LIM-62, University of São Paulo, São Paulo, Brazil; b Department of Oncology, Pain Center, Instituto do Câncer do Estado de São Paulo Octavio Frias de Oliveira, São Paulo, Brazil

The “developing country” categorization has become common in academic settings. There exist, however, several nuances behind these 2 words. There are several ways to classify countries. An exclusively economic classification used by the World Bank ranks countries as high, upper-middle, lower-middle, and low-income states.^14^ This income-based classification is frequently used to differentiate countries of high from low-to-medium income. Differently, the term “developing country” usually refers not only to a state with developed economy and infrastructure, but also takes into account political stability, gross domestic product, freedom, and a high human developing index (HDI). Human developing index is based on life expectancy, education availability, and access to health care. Human developing index ranges from 0 to 1.^6^ Indeed, HDI below 0.8 is customarily used as an operational definition of developing country. There are more than 120 countries currently classified under the developing country umbrella, including nations with large populations such as China, India, and Brazil.^6^ In fact, 4 out of 5 of the 7.7 billion human beings alive today are born, age, and become sick in developing countries. In a way, given these figures, pain occurring in developing countries is the rule rather than the exception in the world.

In the present special issue, Sá et al.^12^ reported the first meta-analysis of chronic pain prevalence studies from developing countries. Reports from Latin America (n = 5), Asia (n = 5), and Africa (n = 2) were included. After adjustment for publication bias, the total prevalence of chronic pain in these regions was estimated to be 18%, similar to previous publications from developed nations. Interestingly, the authors found a significant effect of the definition of chronic pain used in each of these studies on their final reported prevalence. They also found an effect of the year of publication, where later studies had lower chronic pain prevalence rates compared with those published before 2010. Prevalence of pain was also the subject of Machado et al.'s study.^9^ Based on a large multicenter cohort of 15,000 civil servants, the authors reported persistent pain to occur in 62.4% of their sample, being correlated with older age, female sex, excessive drinking, and mood symptoms, among other factors. These data provide insights into the burden of pain in both employed and retired individuals in developing countries and its associated factors.

When it comes to pain relief, developing countries lag behind not only in low access to health care, but also in access to education. Both patient and health care provider education are commonly suboptimal in these regions. Despite the fact that the largest proportion of the world population experiencing pain live in developing countries, health care professionals from these areas have faced several challenges when striving for education. This has been mitigated by several initiatives, such as scholarships to attend congresses, free online educational resources, and low/no subscription fees for professionals from these areas to attend international meetings. However, work remains to be performed. Of nearly 6,000 International Association for the Study of Pain members in 2019, affiliates issuing from developing countries were in the minority: Africa, Latin America, and Middle East (regions mainly composed by developing countries) represented 12% of the Association's members. Similar figures occur in special interest groups, where the proportion of associates from these areas may be below 10% of the total number of members. This proportion discrepancy is also reflected in instances where specific technical or academic credentials are prerequisites to participate, such as for the involvement in international consensuses, task forces, and board activities. In these instances, participation of members from developing countries may range from rare^4^ to absent.^15^ This could lead to a self-fulfilling prophecy where low income leads to low education and low access to good health care from the patient's side. From the provider's end, low access to good quality health care education and research leads to lower representation in high-level scientific and academic settings, which may lead to lower attention to regional particularities by global players and policy makers.

A pragmatic way to start change is to look at one's current medical research environment in a critical way and identify areas that need prioritization. This can be performed in several different ways, one of them being the use of systematic scoping reviews, such as the one reported here by Sharma et al.^13^ Their systematic scoping review covered the state of clinical pain research in Nepal. Sharma et al. found that a very low number of publications assessed pain in children and in the elder. Also, they highlighted a need to develop culturally appropriate outcome measures and to perform cost-effectiveness studies. Such an initiative can provide a roadmap a country can use to organize its efforts for a meaningful and straightforward research program.

Another main challenge is the adaptation of pain assessment tools into the local culture. This was nicely performed by Khampanthip et al.,^7^ who reported cross-cultural adaptation of the Thai version of the University of Washington Pain-Related Self-Efficacy Scale. Pain-related self-efficacy, a person's trust in their ability to tolerate pain and to engage in daily activities despite pain, has close correlations with pain intensity and is also a good predictor of response to pain treatment. This scale has a strong cultural weight, and its formal regional validation is a step forward. Cardona et al.^3^ used a single hospital as a model to gain insights into pediatric pain assessment and management in South Africa. They reported the prevalence of pediatric pain and its assessment in a real-life setting of a tertiary institution, showing that pain was inadequately documented in their pediatric population, despite being very common. They concluded that educational initiatives and the development of regional guidelines were needed.

Even when assessment tools are culturally adapted and high-level research in developing countries can be implemented, new challenges may be revealed as trials start. Parker et al.^10^ described the barriers to implementing trials on nonpharmacological treatments in developing countries, using pain in HIV in South Africa as a model. They showed that loss to follow-up was a main barrier to trial implementation. Contrary to what was thought, loss to follow-up was associated with depressive symptoms and not to sociocultural factors. Thus, the likelihood to remain in a trial may depend on participants' level of depression, which highlights the need to further explore the relationships between pain, suffering, depressive symptoms, and social exclusion.

One could argue poverty and social problems are everywhere, and social inequalities and broader income discrepancies between the richer and the poorer are increasing globally.^16^ Indeed, because classification of countries based on HDI takes into account the average data from a country's population, it misses regional inequalities. Certainly, one can find populations with low HDI living in an overall developed country. This is the case of some native populations and socially excluded groups such as homeless individuals and ethnic minorities living in regions of otherwise developed countries. Arthur et al.^1^ reported a systematic review on western medicine's understanding of Australian Aboriginal and Torres Strait Islander peoples beliefs and attitudes towards pain. The authors reported peculiarities concerning the experience and expression of pain, and its assessment and management in these populations, further addressing discrepancies existing when comparing Australian first peoples and the rest of the country's population in the way pain is interpreted and managed. Campos et al.^2^ assessed pain in people experiencing homelessness on the streets of São Paulo, Brazil. They found that pain in these vulnerable individuals is frequent, being present in higher prevalence than what has been reported in the general population in this same area.^8^ Despite its high prevalence, chronic pain was underdiagnosed and undertreated. The reasons range from low access to health care to low compliance to pharmacological treatment.

It is known that individuals from developing countries face specific societal challenges at a much higher order of magnitude than citizens from developed areas: urban slums, population overgrowth, access to health care, access to clean water and sanitation, and violence against women and the more vulnerable. Each of these factors negatively affects those living with pain in these areas, increasing its burden and suffering. Also, some pain syndromes are particularly highly prevalent in these regions such as pain caused by leprosy, HIV neuropathy, and human T-cell leukemia virus type-1 myelopathy. Sometimes, the term “pain in developing countries” is used to refer to these pain etiologies more frequently found in economically restricted regions. However, in times of massive geographical dislocation, one finds more frequently people issuing from developing countries being treated in developed countries, either by “medical tourism” or by immigration. This increases the need for health care providers from developed countries to be acquainted with diseases previously eradicated from their environment. Moreover, pain related to these diseases is important not only because it affects a large proportion of individuals from developing areas, but in addition, it may provide mechanistic insights into the biology of pain that can be useful to understand the relationship between pain and lesion in other scenarios. For example, intraepidermal nerve fiber density (IENFD) is used to diagnose distal-predominant neuropathy in patients with HIV, especially in those with normal nerve conduction tests. However, because of previous technical standardizations and availability of reference data, skin biopsies are frequently performed over the ankle, irrespective of the presence of pain at this body location. Patel and Kamerman^11^ showed that in patients with neuropathic pain on the feet extending to the ankle, nerve biopsies provided lower intraepidermal nerve fiber density compared with ankle biopsies from patients with pain restricted to the feet. These findings highlighted the importance to couple the site of skin biopsy to the site of neuropathic pain. This is an important observation that, if replicated, may have impact in current recommendations and guidelines on neuropathic pain assessment. Concerning leprosy, Haroun et al.^5^ reported on the somatosensory profile of leprosy neuropathy in India. Besides the novel description of a sensory profile not usually observed in other etiologies of neuropathy (sensory loss to thermal and tactile stimuli combined with preservation of vibration and deep pressure detection), they also reported that impaired thermal cold sensation could be clinically used to enhance identification of leprosy neuropathy at an early stage, when classical field screening of mechanosensation with monofilaments may miss incipient neuropathy. These findings could help better design more accurate bedside sensory testing for use in neuropathic pain assessment in other settings such as in field assessments for other diseases, or even as part of a screening bedside sensory examination protocol meant for general and widespread use by health care providers.

Discussions on pain in developing countries are hard to dissociate from the local social, cultural economic idiosyncrasies of the different countries lumped under this category. Concerted efforts have been made by different pain societies, groups, governmental and nongovernmental associations, and also by single individuals to improve pain-related education in these areas for both providers and for patients. The current situation is certainly better than in the last decades and can be improved further in the years to come.

## Disclosures

The author received grants from CNPq scholarship for research productivity—Brazil; years 2019 to 2021.
